# Different autosomes evolved into sex chromosomes in the sister genera of *Salix* and *Populus*

**DOI:** 10.1038/srep09076

**Published:** 2015-03-13

**Authors:** Jing Hou, Ning Ye, Defang Zhang, Yingnan Chen, Lecheng Fang, Xiaogang Dai, Tongming Yin

**Affiliations:** 1 The Southern Modern Forestry Collaborative Innovation Center, Nanjing Forestry University, Nanjing 210037, China

## Abstract

Willows (*Salix*) and poplars (*Populus*) are dioecious plants in *Salicaceae* family. Sex chromosome in poplar genome was consistently reported to be associated with chromosome XIX. In contrast to poplar, this study revealed that chromosome XV was sex chromosome in willow. Previous studies revealed that both ZZ/ZW and XX/XY sex-determining systems could be present in some species of *Populus*. In this study, sex of *S. suchowensis* was found to be determined by the ZW system in which the female was the heterogametic gender. Gene syntenic and collinear comparisons revealed macrosynteny between sex chromosomes and the corresponding autosomes between these two lineages. By contrast, no syntenic segments were found to be shared between poplar's and willow's sex chromosomes. Syntenic analysis also revealed substantial chromosome rearrangements between willow's alternate sex chromatids. Since willow and poplar originate from a common ancestor, we proposed that evolution of autosomes into sex chromosomes in these two lineages occurred after their divergence. Results of this study indicate that sex chromosomes in *Salicaceae* are still at the early stage of evolutionary divergence. Additionally, this study provided valuable information for better understanding the genetics and evolution of sex chromosome in dioecious plants.

Sex determination has the intriguing aspect in evolutionary and developmental biology. In contrast to the more completed sex chromosome evolution in animals, sex chromosomes in many plants are still at different evolutionary stages^1^,^2^,^3^,^4^, and thus afford the opportunity of investigating the early stage of sex chromosome evolution. It is believed that sex chromosomes originate from a pair of autosomes^5^,^6^, and the sex-determining systems in dioecious plants almost certainly evolved independently from ancestral hermaphrodites that lacked sex chromosomes^2^,^3^. Only about 4% of higher plants show full dioecism, with individuals of separate sexes^7^,^8^,^9^.

*Salicaceae* is a family of dioecious woody plants, and the male or female flowers are arranged in morphologically different catkins (Figure 1) on the male or female trees^10^. *Salix* and *Populus* are the sister genera in *Salicaceae* (Figure 2). Genome analysis revealed that these two lineages originated from a common paleotetrapolyploid ancestor^11^,^12^,^13^. The chromosomes of *Salicaceae* are typically metacentric and small^14^,^15^. Based on cytological studies, there is no evidence of morphologically differentiated sex chromosomes in the *Salicaceae* species^16^,^17^,^18^. Various mechanisms have been proposed to explain the expression of gender in *Salicaceae*. Alström-Rapaport *et al*. proposed a multi-locus sex determination in the *Salix*, and that the presence of sex chromosomes was unlikely^19^. In contrast, Semerikov *et al*. reported that a single locus governed the sex determination and the female was the heterogametic gender in basket willow^20^. More recently, several studies revealed that there was a single locus located on chromosome XIX of *Populus*, with a peritelomeric localization in members of the *Aigeiros* subgenera^21^,^22^ and a centromeric localization in subgenera of *Leuce*^23^,^24^,^25^,^26^, appeared to contain a gene (genes) that controlled gender determination. Multiple lines of evidence suggested that poplar's chromosome XIX was in the process of evolving into an incipient sex chromosome^22^,^27^. The sex-determining region in poplar shows some interesting features, including location of gender locus, highly divergent haplotypes, severe recombination suppression, distinctive pattern of sRNA occurrence, and significantly fewer single nucleotide polymorphisms (SNP) than the rest of *Populus* genome^11^,^22^,^27^. Mapping studies on *P. deltoides*^22^ and *P. alba*^26^ revealed that sex determination occurred through a ZW system in which the female was the heterogametic gender^22^. However, the male sex was reported as the heterogametic gender in *P. nigra*^21^ and *P*. *tremuloides*^23^,^24^,^25^. Thus, it is possible that both ZZ/ZW (female heterogamety) and XX/XY (male heterogamety) sex-determining systems could be present in some members of the family *Salicaceae*^27^. It has been confirmed that the sister genera of *Salix* and *Populus* originate from a common ancestor^11^,^12^,^13^. Since different gender determining systems probably evolve separately and quite recently in species of this family, *Salicaceae* is a desirable system to study the genetics and evolution of sex chromosomes in dioecious plants.

*S. suchowensis* is an early flowering shrub willow that belongs to subgenus *Vetrix*^28^. Recently, its genome has been sequenced and publically available^13^. To gain further insight into the origin and evolution of sex chromosomes in *Salicaceae* species, we identified the sex chromosome in *S. suchowensis*, as well as compared the sex chromosomes between the sister genera of *Salix* and *Populus*.

## Results and Discussion

To map the gender locus of *S. suchowensis*, we established a large mapping pedigree in which the sequenced individual was the maternal parent. Among the progeny, 374 individuals were randomly selected for mapping and locating the gender locus in the willow genome. Investigation of sex phenotype confirmed constant gender for all of the mapping progeny across three locations in two continuous years. Among these individuals, 183 were female, 188 were male, and 3 remained sexually immature. Statistical analysis indicated that segregation of gender fit for the expected 1:1 Mendelian segregation ratio (χ^2^ = 0.067, where (χ^2^ = 3.84 for *α* ≤ 0.05). Subsequently, genetic maps were separately constructed for the maternal and paternal parents by using amplified fragment length polymorphism (AFLP) markers and the pseudo-testcross strategy^29^. Totally, 1,137 1:1 segregating markers were generated by 90 primer combinations with the 374 mapping progeny. Among these markers, 494 testcross markers from the maternal parent were mapped into 266 bins distributed on 19 linkage groups, covering a genetic distance of 1924.5 cM (Supplementary Figure 1); Alternatively, 549 testcross loci from the male were assigned into 263 bins on 19 linkage groups, spanning a genetic distance of 2223.8 cM (Supplementary Figure 2). The coverage of the female and male maps was estimated to be 99.99% and 99.97% at 20 cM of a marker, respectively. Thus, the established maps achieved nearly complete genome coverage, with linkage group numbers equaled to the 19 haploid chromosome numbers in willow. Based on the established genetic maps, the gender locus was mapped as a 1:1 segregating morphological marker. Mapping results showed that gender locus could only be mapped on the maternal map, but was unmappable on the paternal map, indicating that the female was the heterogametic gender in willow, which was in agreement with the findings in *P. deltoides*^22^ and *P. alba*^26^ that revealed sex determination occurred through a ZW system, in which females are heterogametic^22^. It was also noteworthy that marker density in the vicinity of the gender locus (Supplementary Figure 1) was significantly higher than that expected by chance alone (P ≤ 0.0001 with a Poisson calculator), which indicated severe recombination suppression around the gender locus. Recombination suppression has been recognized as a critical mechanism that triggered the divergence of the alternate sex chromosomes^2^,^22^,^30^,^31^.

To map the willow sequence scaffolds derived by Dai *et al*.^13^ along each chromosome of the willow genome, we carried out fine mapping with SNP markers that were generated by resequencing a subset of 80 progeny from the 374 mapping individuals. AFLP markers on the established maps were used as landmarks to anchor the SNP markers into the corresponding marker bins. In total, 2,548 testcross SNP markers from the maternal parent and 3,772 testcross SNP markers from the paternal parent were integrated into the female (Supplementary Figure 3) and male (Supplementary Figure 4) maps, respectively. Subsequently, SNP markers in each linkage group were used to map the willow sequence scaffolds derived by Dai *et al*.^13^ along the corresponding chromosome.

**Figure 3:**
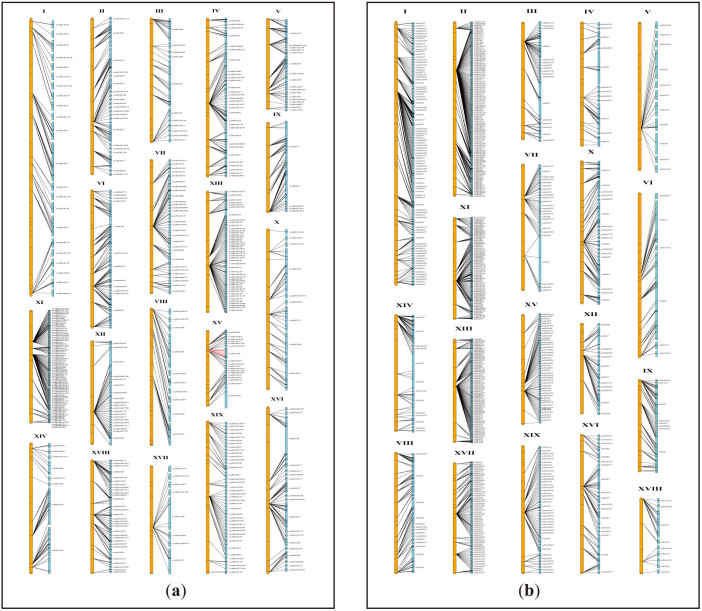
Anchoring the willow sequence scaffolds along each chromosome in the genomes of *Salix suchowensis*. (a) anchor with the female map (b) anchor with the male map. Note: willow sequence scaffolds were obtained from Dai *et al.*'s study^13^. Sequence scaffolds were mapped according to the integrated SNP markers on the maternal genetic map of *S. suchowensis*. The orange bars on the left of each chromosome represented the linkage groups, and the discrete blue bars on the right represented the anchored sequence scaffolds. The mapped scaffolds were separated with evenly sized spaces, which did not represent the actual sizes of the uncaptured gaps. Chromosome identities were designated based on syntenic relationship between the willow's and poplar's chromosomes. As shown in Figure 3a, gender locus was mapped onto scaffold_64 on chromosome XV in the female *S. suchowensis*, and markers cosegregated with gender were indicated with red lines.

In total, 432 sequence scaffolds (covering a physical length of 194.1 Mb) and 630 sequence scaffolds (covering a physical length of 206.2 Mb) were mapped based on the female (Figure 3a) and male (Figure 3b) maps, respectively. There remained 7,009 unmapped sequence scaffolds larger than 2 kb, covering a total physical length of 38.4 Mb. Chromosome identities of the willow genome were designated by blasting willow sequences containing the mapped SNPs against the *P. trichocarpa* genome sequences. It was found that the linkage group containing the gender locus was chromosome XV in the willow genome (Figure 3a). This chromosome is an autosome in poplars. Thus, the willow's sex chromosome corresponds to a poplar's autosome. Previous studies revealed that chromosome XIX was the sex chromosome in poplars^21^,^22^,^23^,^24^,^25^,^26^, by contrast, chromosome XIX was identified as an autosome in willow.

**Figure 4:**
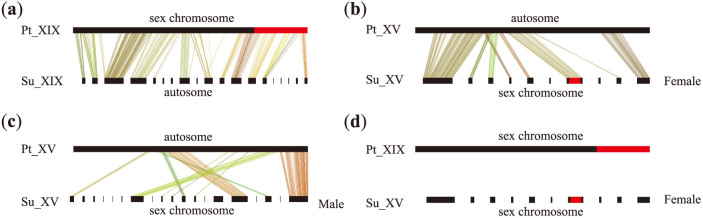
Syntenic analysis between sex chromosomes and the corresponding autosomes across *Populus* *trichocarpa* and *Salix suchowensis*. (a) synteny between poplar's and willow's chromosome XIX based on the female map. (b) synteny between poplar's and willow's chromosome XV based on the female map. (c) synteny between poplar's and willow's chromosome XV based on the male map. (d) synteny between poplar's chromosome XIX and willow's chromosome XV based on the female map. Note: Bar on the top of each chromosome pair represents poplar's chromosome. Bar at the bottom corresponds to the anchored sequence scaffolds on willow's chromosome. Only sequence scaffolds containing coding genes were included in this analysis. The red portions of the chromosome bars represent the sex-determining regions.

On willow's chromosome XV and chromosome XIX, 18 and 40 sequence scaffolds were mapped, respectively, along the corresponding chromosome based on the maternal map (Figure 3a). Because the sequenced individuals of both *P. trichocarpa* and *S. suchowensis* were females^11^,^13^, we first conducted syntenic analysis between sex chromosomes and the corresponding autosomes in these two lineages based on the willow's female map. Syntenic analysis revealed high collinearity on chromosome XIX (Figure 4a) and on chromosome XV (Figure 4b) between willow and poplar. In *P. trichocarpa*, complex haplotype divergence was observed between its alternate sex chromatids^11^,^27^. To explore whether the Z and W sex chromatids diverged with each other in willow, we examined the sequence scaffolds anchored on willow's chromosome XV based on the male map, which included 45 sequence scaffolds (Figure 3b). Contrary to the consistency revealed based on the female map (Figure 4b), syntenic analysis based on the male map revealed substantial chromosome rearrangements (Figure 4c), suggesting the occurrence of significant chromosome divergence between the Z and W chromatids in willow. We further compared willow's sex chromosome (XV) with poplar's sex chromosome (XIX), and found that sex chromosomes between these two lineages shared no syntenic chromosome segments (Figure 4d), indicating that sex chromosomes of poplar and willow originated from different ancestral chromosomes. Since sex chromosomes act as autosomes in the alternate genera of *Salicaceae*, we propose that turnover of autosomes into sex chromosomes occurred after the divergence of *Salix* and *Populus*.

Because evidence indicated that chromosome XV and chromosome XIX separately evolved into sex chromosomes after the divergence of willow and poplar, we proposed that turnover of different autosomes into sex chromosomes might be due to mobile of the sex-determining gene from chromosome XIX to chromosome XV in willow. Alternatively, the sex-determining gene may have translocated from chromosome XV to chromosome XIX in poplar. Therefore, there might be homologous sex-determining genes within the sex-determining regions between willow and poplar. Mapping of SSR markers revealed that the gender locus was in between SSR markers SSR151 (151,741 bp on scaffold 64) and SSR893 (893,817 bp on scaffold 64) (Figure 4b). In *P. nigra*^21^ and *P. deltoides*^22^, the close relatives to *P. trichocarpa* (the sequenced poplar species), gender locus was consistently mapped to the peritelomeric region upper the position of SSR marker O_206 (Figure 4a). We subsequently searched the homologous genes shared by these two regions between willow and poplar, and nine willow genes were found to have a total of 52 homologous genes in the sex-determining region of *P. trichocarpa*. However, all these poplar genes had homologous genes in other regions of the poplar genome, and none of these genes were found to be involved in sex determination based on gene annotation (Supplementary Table 1).

In recent studies, RNA-based sex-determining mechanisms have been found to play a significant role in higher plants^27^,^32^,^33^,^34^. For example, the tasselseed4 miRNA, i.e., miR172, was found to be involved in the sex determination of the male inflorescence in maize^32^,^33^. Interestingly, the miR172 family was conserved in *Populus*^35^ and located on the peritelomeric end of chromosome XIX^27^. We searched the homolgous sequences of miR172 in willow, and none of them appeared in the sex-determining region. More recently, Akagi *et al*. reported that a sex-determining candidate (*OGI*) encoded a small RNA targeting a feminizing gene (*MeGI*) in persimmons (*Diospyros lotus*), and the encoded small RNA acted as a sex determinant in this plant^34^. Search homologous genes of *OGI* and *MeGI* in poplar and willow showed that no homologous genes were found to be distributed on the sex chromosomes in either lineage (Supplementary Table 2). With all of these efforts, we have not identified the likely sex determinants in *Salicaceae*. The involvement of small RNAs in sex determination might explain why they have been difficult to identify to date^34^. RNA-based sex-determining mechanisms maybe present in *Salicaceae* species as well, thus small RNAs will be the primary focus in our future studies.

In plants, species with heterogametic females are less common^36^. Therefore, *Salix* species should represent a unique system for studying dioecy evolution in flowering plants. Although the sex-determining gene (genes) has not been identified with the current data, this work provides unique insight into the sex determination in *Salicaceae* and also sheds new light on understanding the genetics and evolution of sex chromosome in dioecious plants.

## Methods

### Plant materials

An F_1_ mapping pedigree with 2,546 progeny was established in 2010. The maternal parent of this pedigree was described by Dai *et al*.^13^, whereas the paternal parent was a two-year-old *S. suchowensis* collected from Linshu, Shandong Province of China. To map the gender locus, 374 offspring were randomly selected, and cuttings of the selected individuals were planted at three different locations in China: Sihong and Lishui in Jiangsu Province, as well as Shishou in Hubei Province. The field trials were established in 2012, and three replicates were planted for each progeny at each location.

### Map construction

AFLP genotyping was conducted as described by Yin *et al*.^37^. GeneMapper Software (Version 3.7, Applied Biosystems) was used to score the amplicons in range of 50 ~ 500 bp. Map construction, genome length estimation, and map coverage calculation were performed following the description in Yin *et al*.^37^. A Poisson probability test was conducted following the description in Remington *et al*.^38^. The map charts were produced with MapChart 2.1^39^.

### Generating SNP markers

SNP markers were generated with a subset of 80 progeny from the 374 mapping individuals. The combination of two restriction enzymes, EcoRI and MseI (Takara Inc.), were used to reduce the complexity of the willow genome. For PCR amplification, the EcoRI primer had no selective nucleotide, and the MseI primer had a “G” selective nucleotide. The PCR amplification was conducted as described by Yin *et al*.^37^.

The sequencing library was constructed according to the manufacturer's standard protocol (Illumina, Inc.). Library quantification was carried out with a Quant-iTTM PicoGreen dsDNA Kit (Invitrogen, Inc.), and library qualify was evaluated with an Agilent 2100 Bioanalyzer (Agilent, Inc.). For each lane of a flow cell, 12 index-labeled samples were multiplexed, which enabled the sequencing of 84 samples (80 progeny and two replicates of the parents) in a full run, and the eighth lane was used for the control. Sequencing was performed on an Illumina HiSeq 2000 (Illumina, Inc.) at Nanjing Agricultural University following the manufacturer's protocols (Illumina, Inc.).

Raw sequencing data generated by the Illumina platform were analyzed with an integrated bioinformatics pipeline. The SAMTOOLS (http://samtools.sourceforge.net)^40^ and Varscan (http://varscan.source-forge.net) were combined to call the SNPs with default parameters^41^.

### Anchoring the sequence scaffolds

AFLP markers on the established genetic maps were used to pull out tightly linked SNP markers with an LOD threshold ≥10, and these SNPs were assigned to the corresponding marker bins that showed the strongest linkage on each map. Subsequently, the mapped SNPs were used to anchor willow sequence scaffolds that were produced by Dai *et al*.^13^. Finally, willow sequences containing these SNPs were blasted against the *P. trichocarpa* genome sequences (ftp://ftp.jgi-psf.org/pub/compgen/phytozome/v9.0/Ptrichocarpa/) to designate the chromosome identities for the obtained linkage groups on the male and female maps.

### Synteny and collinearity analyses

Genome sequences, Gff3 file, and the protein sequences of *P. trichocarpa* were retrieved from JGI website (ftp://ftp.jgi-psf.org/pub/compgen/phytozo- me/v9.0/Ptrichocarpa/). The corresponding information for willow was downloaded from the *S. suchowensis* website (115.29.234.170/willow). Synteny and collinearity analyses were performed with bioinformatics toolkit of MCScanX^42^. First, coding genes in willow and poplar genome were blasted by using BLASTP^43^ with an e-value cutoff of 1e−10, which produced a file in blast8 format. Then, an in-house perl script was used to filter the gff3 files and reformat the files to make them compatible with MCScanX. Finally, MCScanX produced the collinearity results, with the following parameters: −k 50, −m 25, −e 1e−5, where −k is the match score, −m is the maximum gaps, and −e is the e value cutoff. Dual_synteny_plotter in the MCScanX package was used to draw the collinearity charts.

## Author Contributions

J.H., D.Z. and L.F. conducted the experiments. J.H., N.Y., D.Z. and X.D. performed the data analysis. J.H. and Y.C. prepared the manuscript. T.Y. conceived this work and critically reformulated the manuscript. All authors have read and approved the final manuscript.

## Supplementary Material

Supplementary Information Supplementary information

## Figures and Tables

**Figure 1:**
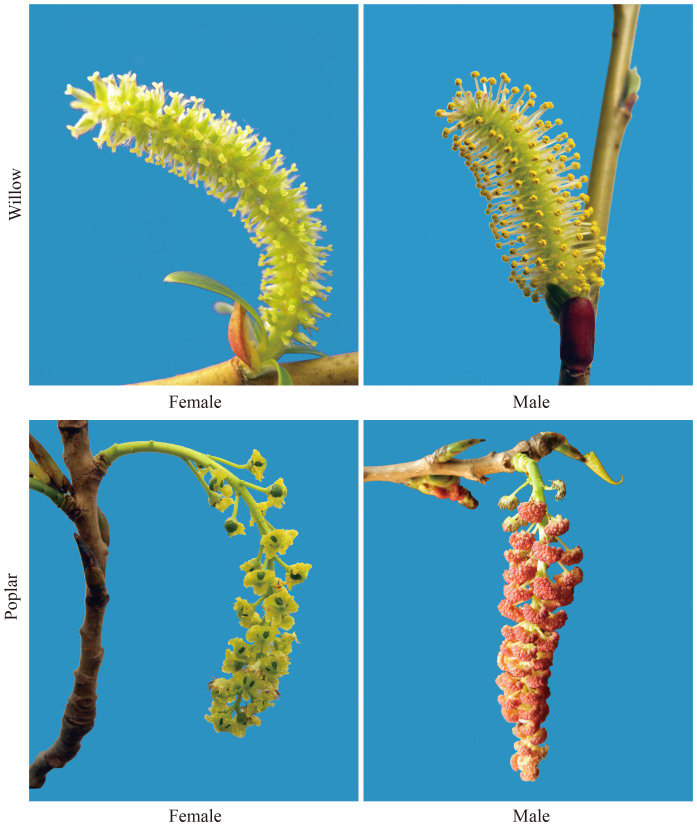
Flowers of the female and male trees in *Salicaceae* species. On willow and poplar, the male or female flowers are separately arranged in morphologically different catkins on the male or female trees. Photos taken by Tongming Yin and Jing Hou.

**Figure 2:**
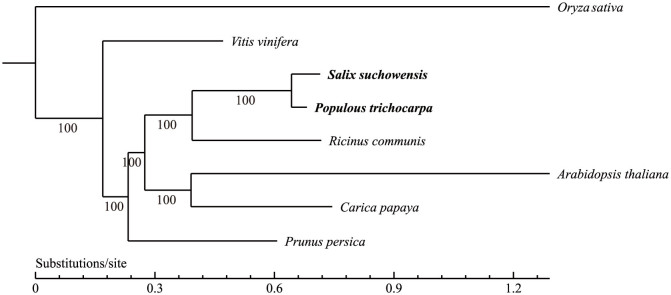
Phylogenetic tree of selected plant species. The phylogenetic tree was constructed with 1,881 single-copy genes on 4-fold degenerate sites. The branch length represents the neutral divergence rate. The posterior probabilities (credibility of the topology) for inner nodes are all 100%.
