# Supplementary material for: Different autosomes evolved into sex chromosomes in the sister genera of Salix and Populus
Source: Sci Rep. 2015 Mar 13;5:9076. doi: 10.1038/srep09076 (PMC4357872; doi:10.1038/srep09076)
Supplement: Supplementary Information [file srep09076-s1.pdf]

Supplementary Information

Different autosomes evolved into sex chromosomes in the sister genera of *Salix* and *Populus*

Jing Hou*, Ning Ye*, Defang Zhang*, Yingnan Chen, Lecheng Fang, Xiaogang Dai, Tongming Yin

The Southern Modern Forestry Collaborative Innovation Center, Nanjing Forestry University, Nanjing 210037, China

To whom correspondence may be addressed:

Dr. Tongming Yin

The Southern Modern Forestry Collaborative Innovation Center,

Nanjing Forestry University,

159#, Longpan Road, Nanjing, 210037, China

Email: tmyin@njfu.com.cn

Phone : 01186-25-85428036

Fax : 01186-25-85427402

Table S1. Homologous genes identified in the gender determining regions (GDR) between poplar and willow and poplar homologous genes in other regions of poplar genome

Homologous genes in the GDR of willow genome	Homologous genes in the GDR of poplar genome	Poplar homologous genes outside the GDR	Gene annotation
willow_GLEAN_10011145	Potri.019G001800	Potri.001G306000;Potri.007G094500;Potri.005G074200;Potri.007G082400	probable receptor-like protein kinase at5g61350
	Potri.019G005300	Potri.019G009800;Potri.019G005900;Potri.019G009700;Potri.019G005700	
	Potri.019G005700	Potri.019G009700;Potri.019G005900;Potri.019G008900;Potri.019G009800	
willow_GLEAN_10011146	Potri.019G004600	Potri.019G004800;Potri.001G308600;Potri.019G005200;Potri.019G009700	probable LRR receptor-like serine threonine-protein kinase at1g74360-like
	Potri.019G004700	Potri.019G005200;Potri.001G308600;Potri.019G006100;Potri.019G007900	
	Potri.019G004800	Potri.001G308600;Potri.019G004600;Potri.019G008900;Potri.019G009700	
	Potri.019G004900	Potri.019G006200;Potri.019G007900;Potri.019G005700;Potri.019G006000	
	Potri.019G005000	Potri.019G005700;Potri.019G007900;Potri.019G006000;Potri.019G005300	
	Potri.019G005200	Potri.001G308600;Potri.019G009700;Potri.019G005900;Potri.019G008900	
	Potri.019G006000	Potri.019G009700;Potri.019G008900;Potri.019G005900;Potri.019G009800	
	Potri.019G006100	Potri.019G009800;Potri.019G005900;Potri.019G009700;Potri.019G005700	
	Potri.019G006200	Potri.019G005300;Potri.019G009800;Potri.019G005900;Potri.019G005700	
	Potri.019G007300	Potri.019G007600;Potri.007G111200;Potri.019G011800;Potri.019G011400	
	Potri.019G007400	Potri.019G011400;Potri.019G007700;Potri.019G011800;Potri.007G111100	
	Potri.019G007600	Potri.019G007300;Potri.007G111200;Potri.019G011800;Potri.019G011400	
	Potri.019G007700	Potri.019G011400;Potri.019G011800;Potri.019G007400;Potri.007G111100	
	Potri.019G007900	Potri.019G008900;Potri.019G005900;Potri.019G005700;Potri.019G009700	
	Potri.019G008900	Potri.019G009700;Potri.019G005900;Potri.019G009800;Potri.019G005700	
	Potri.019G009500	Potri.019G005700;Potri.019G006000;Potri.019G009700;Potri.019G008900	
	Potri.019G009600	Potri.019G009700;Potri.019G008900;Potri.019G005900;Potri.019G005700	
	Potri.019G009700	Potri.019G005900;Potri.019G008900;Potri.019G009800;Potri.019G005700	
	Potri.019G009800	Potri.019G005900;Potri.019G009700;Potri.019G008900;Potri.019G005700	
	Potri.019G011300	Potri.019G011600;Potri.019G007200;Potri.014G107000;Potri.016G104900	
	Potri.019G011400	Potri.019G011800;Potri.019G007700;Potri.019G007400;Potri.007G111100	
	Potri.019G011600	Potri.019G011300;Potri.019G007200;Potri.014G107000;Potri.016G104900	

	Potri.019G011800	Potri.019G011400;Potri.019G007700;Potri.019G007400;Potri.007G111100	
	Potri.019G011900	Potri.019G007600;Potri.019G007300;Potri.007G111200;Potri.005G057000	
	Potri.019G021700	Potri.013G048800;Potri.002G256500;Potri.003G107600;Potri.001G126100	
	Potri.019G025500	Potri.013G051300;Potri.001G053400;Potri.003G174900;Potri.011G139700	
	Potri.019G030600	Potri.013G053000;Potri.006G114400;Potri.002G059000;Potri.005G181800	
willow_GLEAN_10011169	Potri.019G007800	Potri.019G012000;Potri.007G111000;Potri.018G068600;Potri.005G003500	hydrolyzing o-glycosyl
	Potri.019G012000	Potri.019G007800;Potri.007G111000;Potri.018G068600;Potri.005G003500	
willow_GLEAN_10011091	Potri.019G018000	Potri.001G306000;Potri.007G094500;Potri.005G074200;Potri.007G082400	type 2a protein phosphatase-2
willow_GLEAN_10011148	Potri.019G007200	Potri.019G011600;Potri.019G011300;Potri.014G107000;Potri.016G104900	probable serine threonine-protein kinase at1g54610
	Potri.019G018100	Potri.013G046100;Potri.010G071400;Potri.008G166500;Potri.003G120800	
willow_GLEAN_10011105	Potri.019G019200	Potri.002G109500;Potri.013G047000;Potri.019G019300;Potri.005G046000	pentatricopeptide repeat-containing
	Potri.019G019300	Potri.013G047000;Potri.002G109500;Potri.005G046000;Potri.005G038400	
	Potri.019G021200	Potri.006G257300;Potri.004G074700;Potri.006G242500;Potri.006G271400	
	Potri.019G025700	Potri.013G032600;Potri.006G200800;Potri.013G034300;Potri.013G034400	
willow_GLEAN_10011098	Potri.019G020700	Potri.013G047900;Potri.019G020800;Potri.004G091500;Potri.004G091300	probable mitochondrial chaperone bcs1-b-like
	Potri.019G020800	Potri.019G020700;Potri.013G047900;Potri.012G072300;Potri.015G067400	
willow_GLEAN_10011092	Potri.019G025800	Potri.019G026000;Potri.019G025900;Potri.019G026400;Potri.019G026700	germin-like protein subfamily t member 2-like
	Potri.019G025900	Potri.019G026000;Potri.019G025800;Potri.019G026400;Potri.019G026700	
	Potri.019G026000	Potri.019G025900;Potri.019G025800;Potri.019G026400;Potri.019G026700	
	Potri.019G026100	Potri.019G026800;Potri.013G052100;Potri.019G026200;Potri.013G052000	
	Potri.019G026200	Potri.019G026400;Potri.019G026500;Potri.019G026000;Potri.019G025900	
	Potri.019G026400	Potri.019G026700;Potri.019G026000;Potri.019G025900;Potri.019G025800	
	Potri.019G026500	Potri.019G026400;Potri.019G026000;Potri.019G025900;Potri.019G025800	
	Potri.019G026700	Potri.019G026400;Potri.019G026000;Potri.019G025900;Potri.019G025800	
	Potri.019G026800	Potri.019G026100;Potri.013G052100;Potri.019G026200;Potri.013G052000	
	Potri.019G029300	Potri.013G052100;Potri.013G052000;Potri.013G052300;Potri.013G063400	
willow_GLEAN_10011110	Potri.019G033500	Potri.013G059100;Potri.013G059000;Potri.019G033600;Potri.010G056100	short-chain alcohol dehydrogenase

Table S2. Homologous genes of *OGI* and *MeGI* of *Diospyros lotus* identified in poplar and willow.

D. lotus	*Populus trichocarpa*		*Salix suchowenesis*		
**Gene name**	**Gene name**	**Chromosome**	**Gene name**	**Scaffold**	**Chromosome**
KM408638 (*OGI*)	Potri.007G029500	chr7	willow_GLEAN_10010090	scaffold77	chr5
	Potri.005G126100	chr5	willow_GLEAN_10002466	scaffold301	chr7
			willow_GLEAN_10013903	scaffold42	chr7
KM408639	Potri.007G029500	chr7	willow_GLEAN_10010090	scaffold77	chr5
(homolog of *OGI*)	Potri.005G126100	chr5	willow_GLEAN_10013903	scaffold42	chr7
	Potri.002G100600	chr2	willow_GLEAN_10020955	scaffold12	chr2
	Potri.007G097100	chr7			
	Potri.005G071900	chr5			
KM408640	Potri.005G126100	chr5	willow_GLEAN_10010090	scaffold77	chr5
(homolog of *MeGI*)	Potri.007G029500	chr7	willow_GLEAN_10002466	scaffold301	chr7
	Potri.007G097100	chr7	willow_GLEAN_10013903	scaffold42	chr7
	Potri.005G071900	chr5			
	Potri.002G100600	chr2			
KM408641	Potri.007G029500	chr7	willow_GLEAN_10010090	scaffold77	chr5
(homolog of *MeGI*)	Potri.005G126100	chr5	willow_GLEAN_10013903	scaffold42	chr7
	Potri.002G100600	chr2	willow_GLEAN_10020955	scaffold12	chr2
	Potri.007G097100	chr7	willow_GLEAN_10003886	scaffold198	chr5
	Potri.005G071900	chr5	willow_GLEAN_10009523	scaffold82	chr12
	Potri.015G065400	chr15			
	Potri.006G117700	chr6			
KM408642 (*MeGI*)	Potri.007G029500	chr7	willow_GLEAN_10010090	scaffold77	chr5
	Potri.005G126100	chr5	willow_GLEAN_10002466	scaffold301	chr7
	Potri.007G097100	chr7	willow_GLEAN_10013903	scaffold42	chr7
	Potri.005G071900	chr5	willow_GLEAN_10003886	scaffold198	chr5

Note: The genomic sequences of *D. lotus OGI* and *MeGI* (Gene bank IDs KM408638 to KM408642) were refered to Takashi *et al.*[34].

Supplementary figure legends

Figure S1. Genetic map of the maternal parent established with AFLP markers.

Note: Linkage groups (LG) were nominated as those in Figure 3a. Molecular markers were on the left, and marker positions (cM) were on the right of each linkage group. The gender locus was indicated in red bold italic letter on LG XV.

Figure S2. Genetic map of the paternal parent established with AFLP markers.

Note: Linkage groups (LG) were nominated based on Figure 3b. Molecular markers were on the left, and marker positions (cM) were on the right of each linkage group.

Figure S3. Genetic map of maternal parent integrated with the SNPs.

Note: Linkage groups (LG) were nominated as those in Figure 3a. Molecular markers (AFLP and SNP) were on the left, and marker positions (cM) were on the right of each linkage group. The gender locus was indicated in red bold italic letter on LG XV.

Figure S4. Genetic map of paternal parent integrated with the SNPs.

Note: Linkage groups were nominated based on synteny of the integrated SNPs against the *P. trichocarpa* physical map. Molecular markers (AFLP and SNP) were on the left, and marker positions (cM) were on the right of each linkage group.

Figure S1

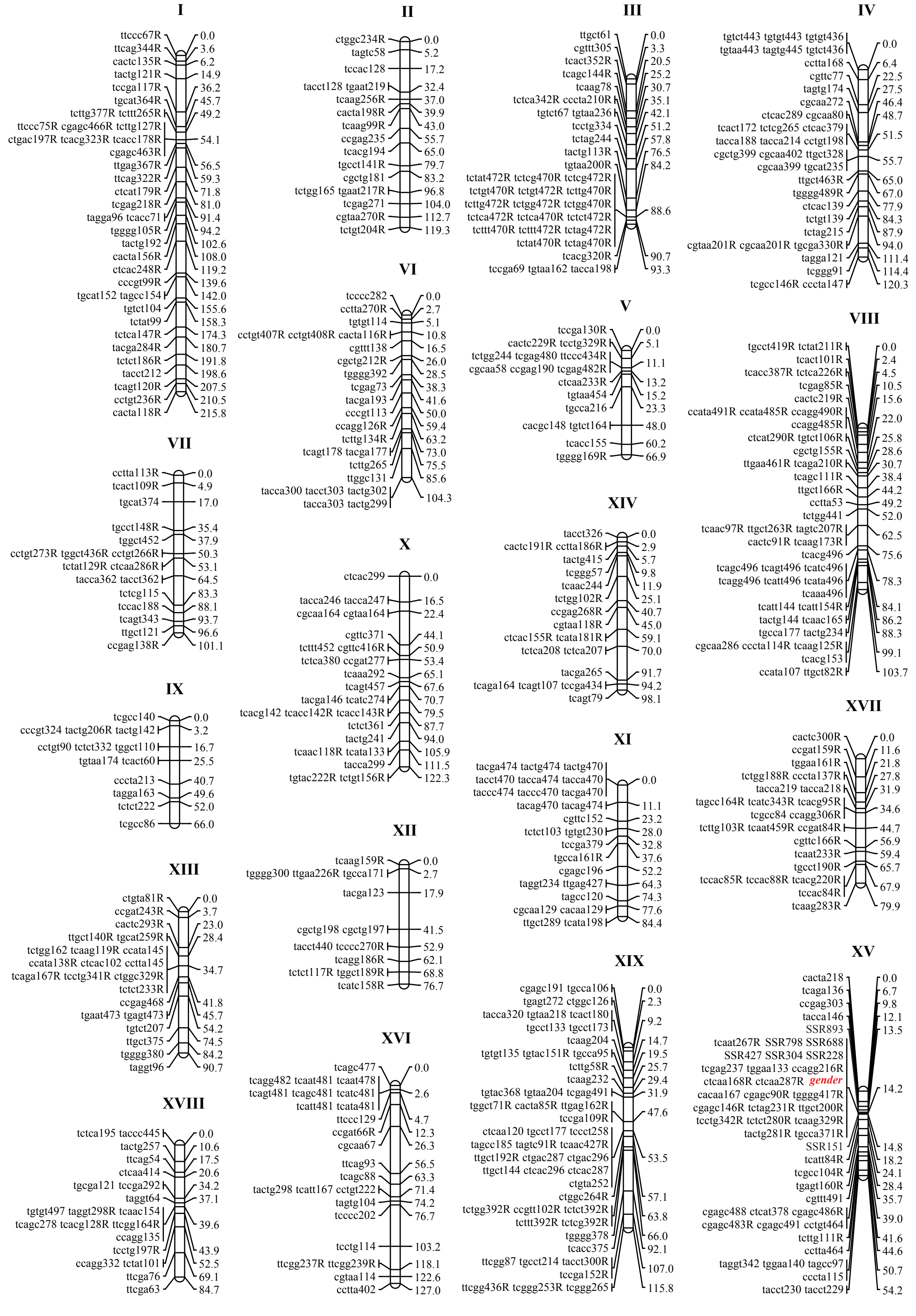
I
ttccc67R 0.0
ttcag344R 3.6
cactc135R 6.2
tactg121R 14.9
tccga117R 36.2
tgcat364R 45.7
tcttg377R tcttt265R 49.2
ttccc75R cgagc466R tcttg127R
ctgac197R tcacg323R tcacc178R 54.1
cgagc463R
ttgag367R 56.5
ttcag322R 59.3
ctcat179R 71.8
tcgag218R 81.0
tagga96 tcacc71 91.4
tgggg105R 94.2
tactg192 102.6
cacta156R 108.0
ctcac248R 119.2
cccgt99R 139.6
tgcat152 tagcc154 142.0
tgtct104 155.6
tctat99 158.3
tctca147R 174.3
tacga284R 180.7
tctct186R 191.8
tacct212 198.6
tcagt120R 207.5
cctgt236R 210.5
cacta118R 215.8
II
ctggc234R 0.0
tagtc58 5.2
tccac128 17.2
tacct128 tgaat219 32.4
tcaag256R 37.0
cacta198R 39.9
tcaag99R 43.0
ccgag235 55.7
tcacg194 65.0
tgcct141R 79.7
cgctg181 83.2
tctgg165 tgaat217R 96.8
tcgag271 104.0
cgtaa270R 112.7
tctgt204R 119.3
III
ttgct61 0.0
cgttt305 3.3
tcact352R 20.5
tcagc144R 25.2
tcaag78 30.7
tctca342R cccta210R 35.1
tgtct67 tgtaa236 42.1
tcctg334 51.2
tctag244 57.8
tactg113R 76.5
tgtaa200R 84.2
tctat472R tctcg470R tctcg472R
tctgt470R tctgt472R tcttg470R
tcttg472R tctgg472R tctgg470R
tctca472R tctca470R tctct472R 88.6
tcttt470R tcttt472R tctag472R
tctat470R tctag470R
tcacg320R 90.7
tccga69 tgtaa162 tacca198 93.3
IV
tgtct443 tgtgt443 tgtgt436
tgtaa443 tagtg445 tgtct436 0.0
cctta168 6.4
cgttc77 22.5
tagtg174 27.5
cgcaa272 46.4
ctcac289 cgcaa80 48.7
tcact172 tctcg265 ctcac379
tacca188 tacca214 cctgt198 51.5
cgctg399 cgcaa402 ttgct328
cgcaa399 tgcat235 55.7
ttgct463R 65.0
tgggg489R 67.0
ctcac139 77.9
tctgt139 84.3
tctag215 87.9
cgtaa201R cgcaa201R tgcga330R 94.0
tagga121 111.4
tcggg91 114.4
tcgcc146R cccta147 120.3
VI
tcccc282 0.0
cctta270R 2.7
tgtgt114 5.1
cctgt407R cctgt408R cacta116R 10.8
cgttt138 16.5
cgctg212R 26.0
tgggg392 28.5
tcgag73 38.3
tacga193 41.6
cccgt113 50.0
ccagg126R 59.4
tcttg134R 63.2
tcagt178 tacga177 73.0
tcttg265 75.5
ttggc131 85.6
tacca300 tacct303 tactg302
tacca303 tactg299 104.3
V
tccga130R 0.0
cactc229R tcctg329R 5.1
tctgg244 tcgag480 ttccc434R 11.1
cgcaa58 ccgag190 tcgag482R
ctcaa233R 13.2
tgtaa454 15.2
tgcca216 23.3
cacgc148 tgtct164 48.0
tcacc155 60.2
tgggg169R 66.9
VIII
tgcct419R tctat211R 0.0
tcact101R 2.4
tcacc387R tctca226R 4.5
tcgag85R 10.5
cactc219R 15.6
ccata491R ccata485R ccagg490R 22.0
ccagg485R
ctcat290R tgtct106R 25.8
cgctg155R 28.6
ttgaa461R tcaga210R 30.7
tcagc111R 38.4
ttgct166R 44.2
cctta53 49.2
tctgg441 52.0
tcaac97R ttgct263R tagtc207R 62.5
cactc91R tcaag173R
tcacg496 75.6
tcagc496 tcagt496 tcatc496
tcagg496 tcatt496 tcata496 78.3
tcaaa496
tcatt144 tcatt154R 84.1
tactg144 tcaac165 86.2
tgcca177 tactg234 88.3
cgcaa286 cccta114R tcaag125R 99.1
tcacg153
ccata107 ttgct82R 103.7
VII
cctta113R 0.0
tcact109R 4.9
tgcat374 17.0
tgcct148R 35.4
tggct452 37.9
cctgt273R tggct436R cctgt266R 50.3
tctat129R ctcaa286R 53.1
tacca362 tacct362 64.5
tctcg115 83.3
tccac188 88.1
tcagt343 93.7
ttgct121 96.6
ccgag138R 101.1
X
ctcac299 0.0
tacca246 tacca247 16.5
cgcaa164 cgtaa164 22.4
cgttc371 44.1
tcttt452 cgttc416R 50.9
tctca380 ccgat277 53.4
tcaaa292 65.1
tcagt457 67.6
tacga146 tcatc274 70.7
tcacg142 tcacc142R tcacc143R 79.5
tctct361 87.7
tactg241 94.0
tcaac118R tcata133 105.9
tacca299 111.5
tgtac222R tctgt156R 122.3
XIV
tacct326 0.0
cactc191R cctta186R 2.9
tactg415 5.7
tcggg57 9.8
tcaac244 11.9
tctgg102R 25.1
ccgag268R 40.7
cgtaa118R 45.0
ctcac155R tcata181R 59.1
tctca208 tctca207 70.0
tacga265 91.7
tcaga164 tcagt107 tccga434 94.2
tcagt79 98.1
IX
tcgcc140 0.0
cccgt324 tactg206R tactg142 3.2
cctgt90 tctct332 tggct110 16.7
tgtaa174 tcact60 25.5
cccta213 40.7
tagga163 49.6
tctct222 52.0
tcgcc86 66.0
XVII
cactc300R 0.0
ccgat159R 11.6
tggaa161R 21.8
tctgg188R cccta137R 27.8
tacca219 tacca218 31.9
tagcc164R tcatc343R tcacg95R
tcgcc84 ccagg306R 34.6
tcttg103R tcaat459R ccgat84R 44.7
cgttc166R 56.9
tcaat233R 59.4
tgcct190R 65.7
tccac85R tccac88R tcacg220R 67.9
tccac84R
tcaag283R 79.9
XI
tacga474 tactg474 tactg470
tacct470 tacca474 tacca470 0.0
taccc474 taccc470 tacga470
tacag470 tacag474 11.1
cgttc152 23.2
tctct103 tgtgt230 28.0
tccga379 32.8
tgcca161R 37.6
cgagc196 52.2
taggt234 ttgag427 64.3
tagcc120 74.3
cgcaa129 cacaa129 77.6
ttgct289 tcata198 84.4
XII
tcaag159R 0.0
tgggg300 ttgaa226R tgcca171 2.7
tacga123 17.9
cgctg198 cgctg197 41.5
tacct440 tcccc270R 52.9
tcagg186R 62.1
tctct117R tggct189R 68.8
tcatc158R 76.7
XIII
ctgta81R 0.0
ccgat243R 3.7
cactc293R 23.0
ttgct140R tgcat259R 28.4
tctgg162 tcaag119R ccata145
ccata138R ctcac102 cctta145 34.7
tcaga167R tcctg341R ctggc329R
tctct233R
ccgag468 41.8
tgaat473 tgagt473 45.7
tgtct207 54.2
ttgct375 74.5
tgggg380 84.2
taggt96 90.7
XIX
cgagc191 tgcca106 0.0
tgagt272 ctggc126 2.3
tacca320 tgtaa218 tcact180 9.2
tgcct133 tgcct173
tcaag204 14.7
tgtgt135 tgtac151R tgcca95 19.5
tcttg58R 25.7
tcaag232 29.4
tgtac368 tgtaa204 tcgag491 31.9
tggct71R cacta85R ttgag162R 47.6
tccga109R
ctcaa120 tgcct177 tccct258
tagcc185 tagtc91R tcaac427R
ttgct192R ctgac287 ctgac296 53.5
ttgct144 ctcac296 ctcac287
ctgta252
ctggc264R 57.1
tctgg392R ccgtt102R tctct392R 63.8
tcttt392R tcctg392R
tgggg378 66.0
tcacc375 92.1
ttcgg87 tgcct214 tacct300R 107.0
tccga152R
ttcgg436R tcggg253R tcggg265 115.8
XV
cacta218 0.0
tcaga136 6.7
ccgag303 9.8
tacca146 12.1
SSR893 13.5
tcaat267R SSR798 SSR688
SSR427 SSR304 SSR228
tcgag237 tggaa133 ccagg216R
ctcaa168R ctcaa287R gender 14.2
cacaa167 cgagc90R tgggg417R
cgagc146R tctag231R ttgct200R
tcctg342R tctct280R tcaag329R
tactg281R tgcca371R
SSR151 14.8
tcatt84R 18.2
tcgcc104R 24.1
tgagt160R 28.4
cgttt491 35.7
cgagc488 ctcat378 cgagc486R 39.0
cgagc483R cgagc491 cctgt464
tcttg111R 41.6
cctta464 44.6
taggt342 tggaa140 tagcc97 50.7
cccta115
tacct230 tacct229 54.2
XVIII
tctca195 taccc445 0.0
tactg257 10.6
ttcag54 17.5
ctcaa414 20.6
tgcga121 tccga292 34.2
taggt64 37.1
tgtgt497 taggt298R tcaac154
tcagc278 tcacg128R ttcgg164R 39.6
ccagg135
tcctg197R 43.9
ccagg332 tctat101 52.5
ttcga76 69.1
ttcga63 84.7
XVI
tcagc477 0.0
tcagg482 tcaat481 tcaat478
tcagt481 tcagc481 tcatc481 2.6
tcatt481 tcata481
ttccc129 4.7
ccgat66R 12.3
cgcaa67 26.3
ttcag93 56.5
tcagc88 63.3
tactg298 tcatt167 cctgt222 71.4
tagtg104 74.2
tcccc202 76.7
tcctg114 103.2
ttcgg237R ttcgg239R 118.1
cgtaa114 122.6
cctta402 127.0

Figure S2

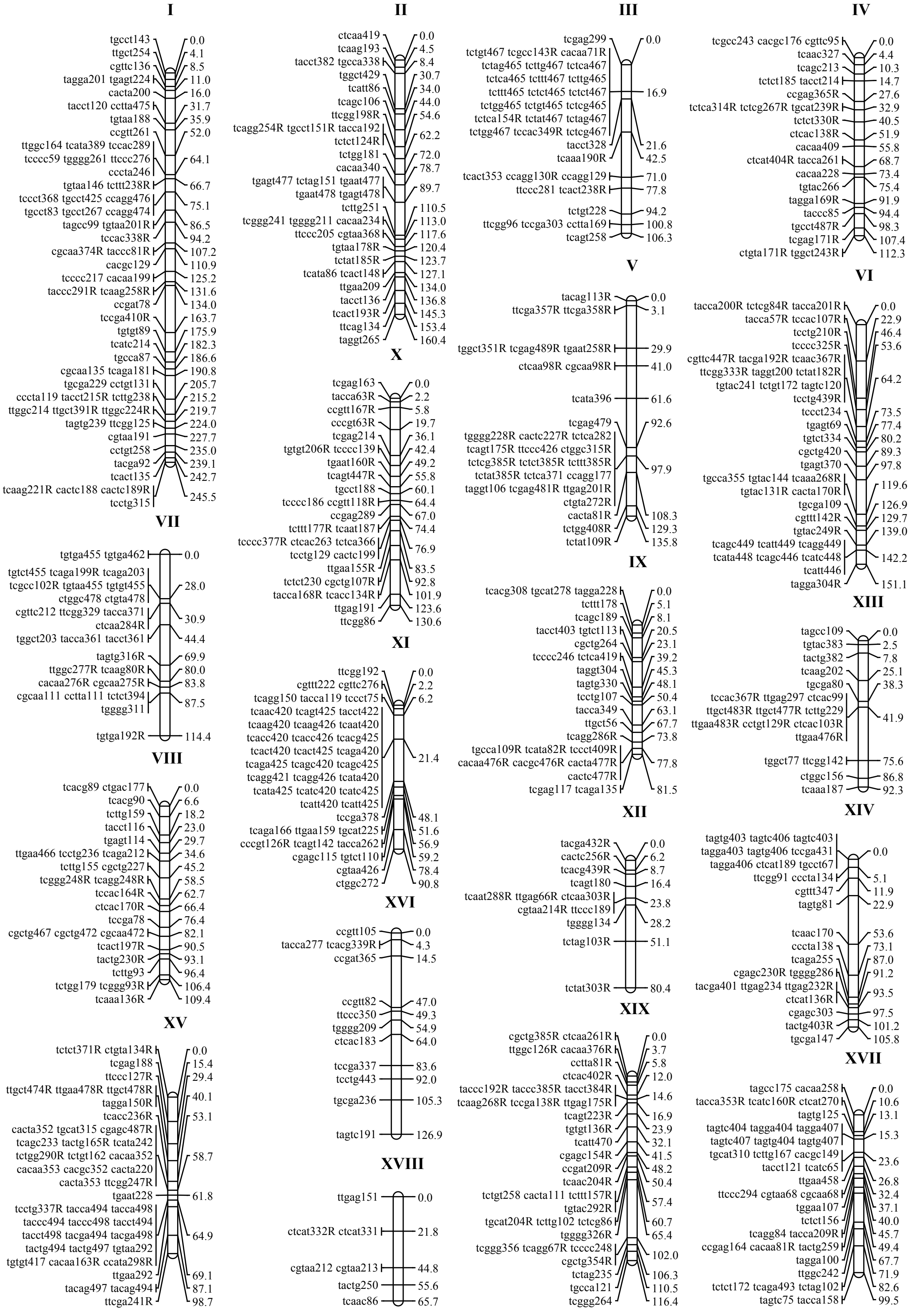
I
tgcct143
ttgct254
cgttc136
tagga201 tgagt224
cacta200
tacct120 cctta475
tgtaa188
ccgtt261
ttggc164 tcata389 tccac289
tcccc59 tgggg261 ttccc276
cccta246
tgtaa146 tcttt238R
tccct368 tgcct425 ccagg476
tgcct83 tgcct267 ccagg474
tagcc99 tgtaa201R
tccac338R
cgcaa374R taccc81R
cacgc129
tcccc217 cacaa199
taccc291R tcaag258R
ccgat78
tccga410R
tgtgt89
tcatc214
tgcca87
cgcaa135 tcaga181
tgcga229 cctgt131
cccta119 tacct215R tcttg238
ttggc214 ttgct391R ttggc224R
tagtg239 ttcgg125
cgtaa191
cctgt258
tacga92
tcact135
tcaag221R cactc188 cactc189R
tcctg315
0.0
4.1
8.5
11.0
16.0
31.7
35.9
52.0
64.1
66.7
75.1
86.5
94.2
107.2
110.9
125.2
131.6
134.0
163.7
175.9
182.3
186.6
190.8
205.7
215.2
219.7
224.0
227.7
235.0
239.1
242.7
245.5
II
ctcaa419
tcaag193
tacct382 tgcca338
tggct429
tcatt86
tcagc106
ttcgg198R
tcagg254R tgcct151R tacca192
tctct124R
tctgg181
cacaa340
tgagt477 tctag151 tgaat477
tgaat478 tgagt478
tcttg251
tcggg241 tgggg211 cacaa234
ttccc205 cgtaa368
tgtaa178R
tctat185R
tcata86 tcact148
ttgaa209
tacct136
tcact193R
ttcag134
taggt265
0.0
4.5
8.4
30.7
34.0
44.0
54.6
62.2
72.0
78.7
89.7
110.5
113.0
117.6
120.4
123.7
127.1
134.0
136.8
145.3
153.4
160.4
III
tcgag299
tctgt467 tcgcc143R cacaa71R
tctag465 tcttg467 tctca467
tctca465 tcttt467 tcttg465
tcttt465 tctct465 tctct467
tctgg465 tctgt465 tctcg465
tctca154R tctat467 tctag467
tctgg467 tccac349R tctcg467
tacct328
tcaaa190R
tcact353 ccagg130R ccagg129
ttccc281 tcact238R
tctgt228
ttcgg96 tccga303 cctta169
tcagt258
0.0
16.9
21.6
42.5
71.0
77.8
94.2
100.8
106.3
IV
tcgcc243 cacgc176 cgttc95
tcaac327
tcagc213
tctct185 tacct214
ccgag365R
tctca314R tctcg267R tgcat239R
tctct330R
ctcac138R
cacaa409
ctcat404R tacca261
cacaa228
tgtac266
tagga169R
taccc85
tgcct487R
tcgag171R
ctgta171R tggct243R
0.0
4.4
10.3
14.7
27.6
32.9
40.5
51.9
55.8
68.7
73.4
75.4
91.9
94.4
98.3
107.4
112.3
V
tacag113R
ttcga357R ttcga358R
tggct351R tcgag489R tgaat258R
ctcaa98R cgcaa98R
tcata396
tcgag479
tgggg228R cactc227R tctca282
tcagt175R ttccc426 ctggc315R
tctcg385R tctct385R tcttt385R
tctat385R tctca371 ccagg177
taggt106 tcgag481R ttgag201R
ctgta272R
cacta81R
tctgg408R
tctat109R
0.0
3.1
29.9
41.0
61.6
92.6
97.9
108.3
129.3
135.8
VI
tacca200R tctcg84R tacca201R
tacca57R tccac107R
tcctg210R
tcccc325R
cgttc447R tacga192R tcaac367R
ttcgg333R taggt200 tctat182R
tgtac241 tctgt172 tagtc120
tcctg439R
tccct234
tgagt69
tgtct334
cgctg420
tgagt370
tgcca355 tgtac144 tcaaa268R
tgtac131R cacta170R
tgcga109
cgttt142R
tgtac249R
tcagc449 tcatt449 tcagg449
tcata448 tcagc446 tcatc448
tcatt446
tagga304R
0.0
22.9
46.4
53.6
64.2
73.5
77.4
80.2
89.3
97.8
119.6
126.9
129.7
139.0
142.2
151.1
VII
tgtga455 tgtga462
tgtct455 tcaga199R tcaga203
tcgcc102R tgtaa455 tgtgt455
ctggc478 ctgta478
cgttc212 ttcgg329 tacca371
ctcaa284R
tggct203 tacca361 tacct361
tagtg316R
ttggc277R tcaag80R
cacaa276R cgcaa275R
cgcaa111 cctta111 tctct394
tgggg311
tgtga192R
0.0
28.0
30.9
44.4
69.9
80.0
83.8
87.5
114.4
VIII
tcacg89 ctgac177
tcacg90
tcttg159
tacct116
tgagt114
ttgaa466 tcctg236 tcaga212
tcttg155 cgctg227
tcggg248R tcagg248R
tccac164R
ctcac170R
tccga78
cgctg467 cgctg472 cgcaa472
tcact197R
tactg230R
tcttg93
tctgg179 tcggg93R
tcaaa136R
0.0
6.6
18.2
23.0
29.7
34.6
45.2
58.5
62.7
66.4
76.4
82.1
90.5
93.1
96.4
106.4
109.4
IX
tcacg308 tgcat278 tagga228
tcttt178
tcagc189
tacct403 tgtct113
cgctg264
tcccc246 tctca419
taggt304
tagtg330
tcctg107
tacca349
ttgct56
tcagg286R
tgcca109R tcata82R tccct409R
cacaa476R cacgc476R cacta477R
cactc477R
tcgag117 tcaga135
0.0
5.1
8.1
20.5
23.1
39.2
45.3
48.1
50.4
63.1
67.7
73.8
77.8
81.5
X
tcgag163
tacca63R
ccgtt167R
cccgt63R
tcgag214
tgtgt206R tcccc139
tgaat160R
tcagt447R
tgcct188
tcccc186 ccgtt118R
ccgag289
tcttt177R tcaat187
tcccc377R ctcac263 tctca366
tcctg129 cactc199
ttgaa155R
tctct230 cgctg107R
tacca168R tcacc134R
ttgag191
ttcgg86
0.0
2.2
5.8
19.7
36.1
42.4
49.2
55.8
60.1
64.4
67.0
74.4
76.9
83.5
92.8
101.9
123.6
130.6
XI
ttcgg192
cgttt222 cgttc276
tcagg150 tacca119 tccct75
tcaac420 tcagt425 tacct422
tcaag420 tcaag426 tcaat420
tcacc420 tcacc426 tcacg425
tcact420 tcact425 tcaga420
tcaga425 tcagc420 tcagc425
tcagg421 tcagg426 tcata420
tcata425 tcatc420 tcatc425
tcatt420 tcatt425
tccga378
tcaga166 ttgaa159 tgcat225
cccgt126R tcagt142 tacca262
cgagc115 tgtct110
cgtaa426
ctggc272
0.0
2.2
6.2
21.4
48.1
51.6
56.9
59.2
78.4
90.8
XII
tacga432R
cactc256R
tcacg439R
tcagt180
tcaat288R ttgag66R ctcaa303R
cgtaa214R ttccc189
tgggg134
tctag103R
tctat303R
0.0
6.2
8.7
16.4
23.8
28.2
51.1
80.4
XIII
tagcc109
tgtac383
tactg382
tcaag202
tgcga80
tccac367R ttgag297 ctcac99
ttgct483R ttgct477R tcttg229
ttgaa483R cctgt129R ctcac103R
ttgaa476R
tggct77 ttcgg142
ctggc156
tcaaa187
0.0
2.5
7.8
25.1
38.3
41.9
75.6
86.8
92.3
XIV
tagtg403 tagtc406 tagtc403
tagga403 tagtg406 tccga431
tagga406 ctcat189 tgcct67
ttcgg91 cccta134
cgttt347
tagtg81
tcaac170
cccta138
tcaga255
cgagc230R tgggg286
tacga401 ttgag234 ttgag232R
ctcat136R
cgagc303
tactg403R
tgcga147
0.0
5.1
11.9
22.9
53.6
73.1
87.0
91.2
93.5
97.5
101.2
105.8
XV
tctct371R ctgta134R
tcgag188
ttccc127R
ttgct474R ttgaa478R ttgct478R
tagga150R
tcacc236R
cacta352 tgcat315 cgagc487R
tcagc233 tactg165R tcata242
tctgg290R tctgt162 cacaa352
cacaa353 cacgc352 cacta220
cacta353 ttcgg247R
tgaat228
tcctg337R tacca494 tacca498
taccc494 taccc498 tacct494
tacct498 tacga494 tacga498
tactg494 tactg497 tgtaa292
tgtgt417 cacaa163R ccata298R
ttgaa292
tacag497 tacag494
ttcga241R
0.0
15.4
29.4
40.1
53.1
58.7
61.8
64.9
69.1
87.1
98.7
XVI
ccgtt105
tacca277 tcacg339R
ccgat365
ccgtt82
ttccc350
tgggg209
ctcac183
tccga337
tcctg443
tgcga236
tagtc191
0.0
4.3
14.5
47.0
49.3
54.9
64.0
83.6
92.0
105.3
126.9
XVII
tagcc175 cacaa258
tacca353R tcatc160R ctcat270
tagtg125
tagtc404 tagga404 tagga407
tagtc407 tagtg404 tagtg407
tgcat310 tcttg167 cacgc149
tacct121 tcatc65
ttgaa458
ttccc294 cgtaa68 ccgaa68
tggaa107
tctct156
tcagg84 tacca209R
ccgag164 cacaa81R tactg259
tagga100
ttggc242
tctct172 tcaga493 tctag102
tagtc75 tacca158
0.0
10.6
13.1
15.3
23.6
26.8
32.4
37.1
40.0
45.7
49.4
67.7
71.9
82.6
99.5
XVIII
ttgag151
ctcat332R ctcat331
cgtaa212 cgtaa213
tactg250
tcaac86
0.0
21.8
44.8
55.6
65.7
XIX
cgctg385R ctcaa261R
ttggc126R cacaa376R
cctta81R
ctcac402R
taccc192R taccc385R tacct384R
tcaag268R tccga138R ttgag175R
tcagt223R
tgtgt136R
tcatt470
cgagc154R
ccgat209R
tcaac204R
tctgt258 cacta111 tcttt157R
tgtac292R
tgcat204R tcttg102 tctcg86
tgggg326R
tcggg356 tcagg67R tcccc248
cgctg354R
tctag235
tgcca121
tcggg264
0.0
3.7
5.8
12.0
14.6
16.9
23.9
32.1
41.5
48.2
50.4
57.4
60.7
65.4
102.0
106.3
110.5
116.4

Figure S3

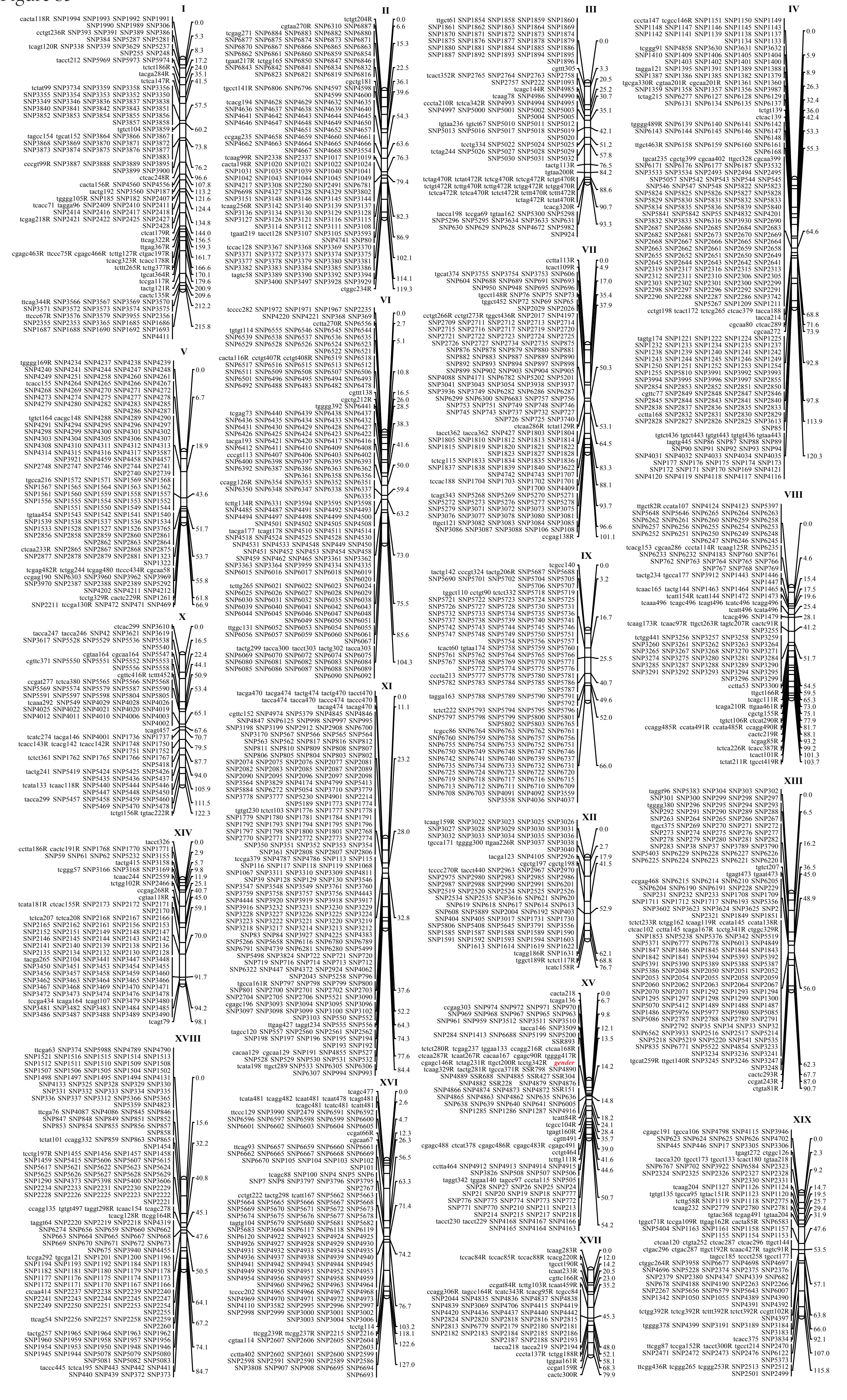
I
II
III
IV
V
VI
VII
VIII
IX
X
XI
XII
XIII
XIV
XV
XVI
XVII
XVIII
XIX

Figure S4

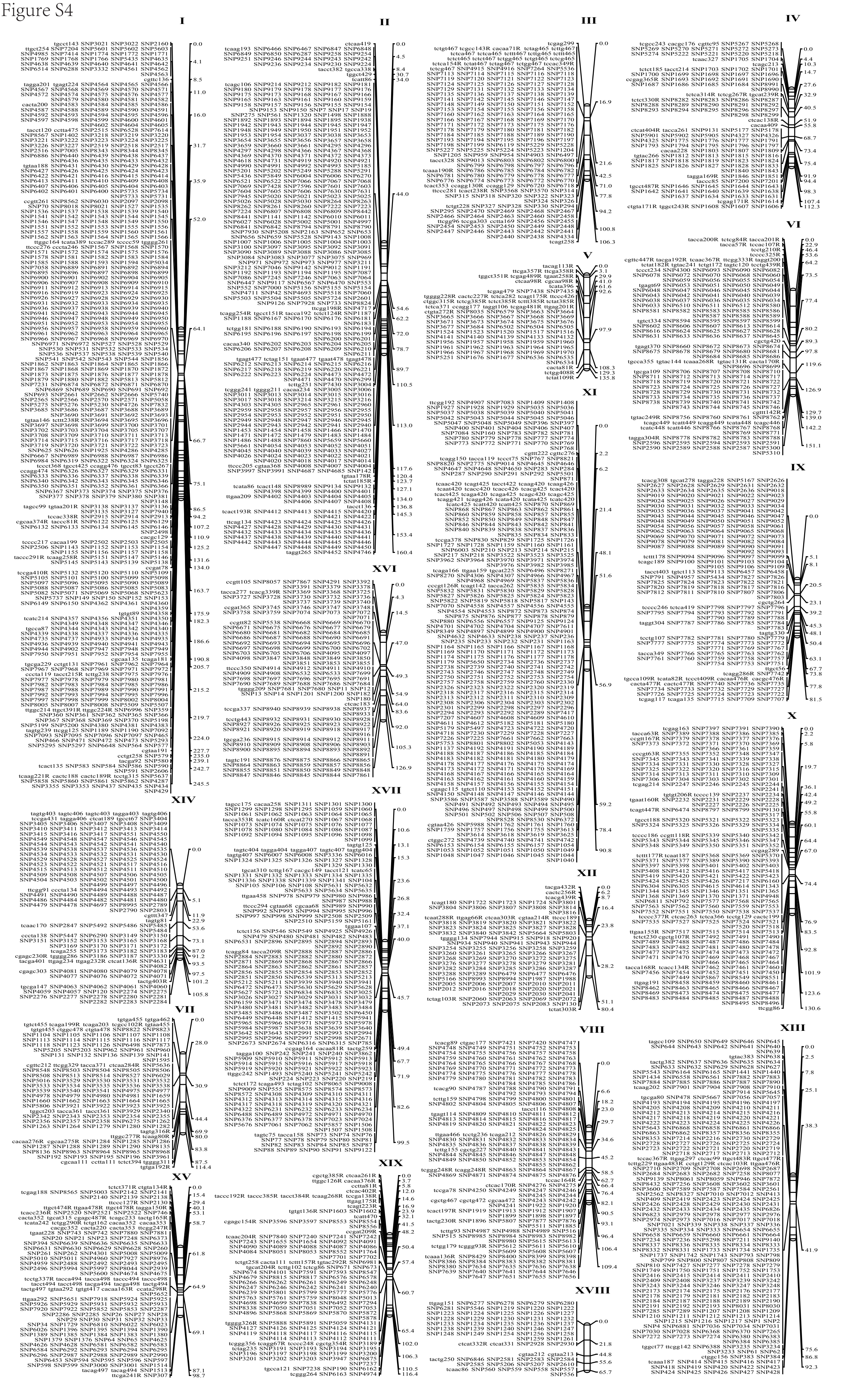
I
II
III
IV
V
VI
VII
VIII
IX
X
XI
XII
XIII
XIV
XV
XVI
XVII
XVIII
XIX
